# Impact of immunosuppressive regimens on antibody response after COVID-19 vaccination among Thai kidney transplant recipients

**DOI:** 10.1016/j.heliyon.2025.e42291

**Published:** 2025-01-25

**Authors:** Nuttasith Larpparisuth, Kritsada Pongsakornkullachart, Nartsiri Ratchawang, Attapong Vongwiwatana, Peenida Skulratanasak

**Affiliations:** a Division of Nephrology, Department of Medicine, Faculty of Medicine Siriraj Hospital, Mahidol University, Bangkok, Thailand; b Department of Nursing Siriraj Hospital, Faculty of Medicine Siriraj Hospital, Mahidol University, Bangkok, Thailand

**Keywords:** SARS-CoV-2, COVID-19, Kidney transplantation, ChAdOx1 nCoV-19 vaccine, Anti-Spike RBD IgG

## Abstract

**Background:**

The lower humoral immunity response after the COVID-19 vaccine in kidney transplant recipients (KTR) has been reported in several studies. However, there are few studies on the efficacy of the ChAdOx1 nCoV-19 (AstraZeneca) vaccine compared between various immunosuppressive regimens.

**Methods:**

We conducted a prospective cohort study at Siriraj Hospital, Bangkok, Thailand. Adult KTRs who received two doses of the ChAdOx1 nCoV-19 vaccine at intervals of 3 months were enrolled. Anti-SARS-COV-2 S-RBD-IgG antibody (anti-RBD) was assessed at the one month after the second dose and considered positive if the level ≥50 AU/mL or 7 BAU/mL. The primary outcome was the seropositivity of anti-RBD. The association between type, dose, and level of immunosuppressive regimen and anti-RBD seropositivity was analyzed.

**Results:**

Between October 2021 and January 2022, 139 KTRs with a median time of 55 months (IQR, 29–102 months), were enrolled. The mean age was 49.1 ± 11.3 years and 64.7 % were men. Seroconversion of anti-RBD was found in 72 patients (51.8 %). The seropositive rate was significantly higher in KTR who received tacrolimus (TAC)/everolimus (EVR)/prednisolone (CS) immunosuppression than EVR/mycophenolic acid (MPA)/CS and TAC/MPA/CS, respectively (95 % vs. 65 % vs. 34 %; *p* < 0.001). The MPA-containing regimen is associated with an inferior humoral response (OR 0.02, 95%CI 0.01–0.16; *p* < 0.001). In contrast, KTRs who received EVR had the highest immunogenic response (OR 12.97, 95%CI 4.69–35.84; *p* < 0.001). During the 11-month follow-up period, COVID-19 pneumonia occurred in 3 KTR in the seronegative group and none in the seropositive group.

**Conclusion:**

The anti-RBD response after ChAdOx1 nCoV-19 vaccination was revealed in 51.8 % of the KTR. KTRs who received the TAC/EVR/CS regimen had the highest immune response after vaccination, relatively comparable to the general population. The immunosuppressive regimen should be considered for a further vaccine dose in KTR.

## 1 Introduction

Coronavirus disease 2019 (COVID-19) is an infectious disease caused by the severe acute respiratory syndrome coronavirus 2 (SARS-CoV-2) virus. Kidney transplant recipients (KTR) are particularly vulnerable to severe COVID-19, including pneumonia and multi-organ failure [1]. Studies have shown that KTRs face a higher morbidity and mortality rate from COVID-19 compared to both the general population and dialysis patients [2]. Therefore, COVID-19 vaccination is strongly recommended for KTRs [3]. However, the immunologic response to vaccination, including for hepatitis B virus, influenza, or herpes zoster vaccines, is significantly lower in KTRs compared to both healthy individuals and patients with chronic kidney disease (CKD) [4,5].

Seroconversion rate following COVID-19 vaccination has shown considerable variability, ranging from 54.8 % to 97.6 %, depending on the vaccine platform and the specific population, with suboptimal responses observed among transplant patients [[6], [7], [8]]. Vaccination in KTRs is a challenging issue due to the diminished immune response post-vaccination caused by the immunosuppressive regimen [[9], [10], [11]]. The standard immunosuppressive protocol for KTRs typically includes tacrolimus (TAC), mycophenolic acid (MPA), and prednisolone. However, in case of calcineurin inhibitor nephrotoxicity, viral infection and/or malignancy post transplantation, substitution of TAC or MPA with a mammalian target of rapamycin inhibitor (mTORi), such as sirolimus or everolimus, may be considered. Periodic withdrawal of immunosuppressants during the peri-vaccination period has been proposed to enhance seroconversion rates, particularly in patients with autoimmune diseases [12]. However, intermittent cessation of immunosuppressants in KTRs carries the risk of kidney allograft rejection and subsequent graft loss [13].

In Thailand, the COVID-19 vaccination program commenced in March 2021 with a focus on prioritizing transplant patients [14]. During the initial phase of the vaccination rollout, the primary vaccine administered to transplant patients was the ChAdOx1 nCoV-19 vaccine (AstraZeneca), which comprises a replication-deficient chimpanzee adenoviral vector, ChAdOx1, containing the SARS-CoV-2 spike protein (nCoV-19) [15]. Limited information was available regarding the immune response following ChAdOx1 nCoV-19 vaccination, particularly among KTRs. Therefore, this study aimed to assess the immunogenicity of ChAdOx1 nCoV-19 vaccine by evaluating the post-vaccination levels of anti-SARS-CoV-2 spike receptor-binding protein immunoglobulin G antibody (anti-RBD) in KTRs receiving various immunosuppressive regimen.

## 2 Methods

We conducted a single-center prospective observational study at Siriraj Hospital, Mahidol University, Bangkok, Thailand. This study was approved by the Ethics Committee of Siriraj Hospital Faculty of Medicine, Mahidol University, Bangkok, Thailand (COA no. Si 928/2021). Adults (≥18 years old) who had undergone kidney transplantation (KT) more than 3 months prior, with stable kidney function, and had received two doses of the ChAdOx1 nCoV-19 vaccine from their local clinic or hospital, were recruited. All participants received the two doses of ChAdOx1 nCoV-19 vaccine with a 12-week interval as per the routine schedule, as recorded in the mobile application of the Thai Ministry of Public Health. Exclusion criteria included: 1) prior SARS-CoV-2 infection, as defined by self-reported symptomatic infection; 2) receipt other types of COVID-19 vaccines; 3) combined organ transplantation, except simultaneous pancreas-kidney (SPK) transplantation; 4) recent treatment for allograft rejection; and 5) failure to provide informed consent. Our focus was on three immunosuppression regimens, including 1) tacrolimus (TAC), mycophenolic acid (MPA), and prednisolone (CS) (referred to as the standard regimen); 2) everolimus (EVR), MPA and CS regimens; and 3) TAC, EVR and CS regimen.

Eligible participants were evaluated and informed consent was obtained. The baseline characteristics of the study participants, including comorbidities, KT data, and immunosuppressive regimens, were collected. The two doses of ChAdOx1 nCoV-19 vaccine with a 3-month interval as the routine schedule was prescribed to all participants. Serum anti-RBD was evaluated four weeks after the second dose of the vaccine. An anti-RBD was measured using Abbott's SARS-CoV-2 IgG II quantitative antibody assay, which was performed using chemiluminescent microparticle immunoassay (CMIA) technique. This assay quantitatively measures immunoglobulin G (IgG) antibodies against the spike receptor binding domain (RBD) of SARS-CoV-2 [16,17]. A seropositive result is defined as an anti-RBD level ≥50 arbitrary units per milliliter (AU/mL) which is equivalent to 7 binding antibody units per milliliter (BAU/mL).

All participants were monitored for symptomatic SARS-CoV-2 infection over a 12-month period after receiving the second dose of the ChAdOx1 nCoV-19 vaccine. Infection was confirmed by detecting viral RNA using the real-time polymerase chain reaction (RT-PCR) method from nasopharyngeal swabs [18]. For infected participants, the severity of COVID-19 infection was recorded. Seronegative participants who received an additional dose of the SARS-CoV-2 vaccine were scheduled for a second anti-spike RBD IgG test after the third dose of vaccination. All data were analyzed to determine the relationship between the results of the anti-spike RBD IgG test after the second dose of the ChAdOx1 nCoV-19 vaccine and maintenance immunosuppression.

## 3 Statistical analysis

Baseline characteristics are shown as percentage (%), number and percentage (%), or mean ± standard deviation (SD) for normally distributed data, and as median and interquartile range (IQR) for data with skewed distribution. Categorical variables were compared using the Pearson Chi-square or Fisher's exact test. Continuous variables were compared using Student's *t*-test or Mann-Whitney *U* test according to the data distribution. Univariate and multivariate logistic regression analysis was used to identify factors associated with seroconversion after vaccination and were reported as odd ratio (OR) and 95 % confidence interval. Statistical significance is defined as a two-tailed *p*-value <0.05. All statistical analyzes were performed with SPSS version 18.0 (IBM, Armonk, NY, USA).

## 4 Results

Between October 2021 and January 2022, 140 KTRs were screened and enrolled in our study. Only 1 KTRs were excluded due to incomplete antibody testing. The remaining 139 KTRs, with a median time after KT of 55 (29–102) months were analyzed (Supplementary Fig. 1). Baseline characteristics of patients stratified by anti-spike RBD IgG status after vaccination are shown in Table 1. The mean age was 49.1 ± 11.3 years, and 90 patients (64.7 %) were men. Most of the patients (81 patients, 58.3 %) underwent KT from a deceased donor. Most patients (66.2 %) received a standard immunosuppressive regimen (TAC + MPA + CS; Regimen 1), while 24 (17.3 %) and 23 (16.5 %) patients received TAC + EVR + CS (Regimen 2) and EVR + MPA + CS (Regimen 3), respectively. The dose of immunosuppressive medications and the TAC and EVR trough levels are shown in Supplementary Tables 1 and 2

**Table 1:** Clinical characteristics of KTRs according to anti-spike RBD IgG status after receiving two doses of the ChAdOx1 nCoV-19 vaccine.

Characteristics		Total (N = 139)	Anti-spike RBD IgG Level		
			Seropositive (N = 72)	Seronegative (N = 67)	*p-value*
**Mean age (SD) – year**		49.1 (11.3)	48.0 (11.5)	50.3 (10.4)	0.23
**Male - number of participants (%)**		90 (64.7)	48 (66.7)	42 (62.7)	0.62
**Time from transplantation - median (IQR), months**		55 (29–102)	54 (30–101)	59 (26–103)	0.75
**Types of transplants - number of participants (%)**					
Deceased donor kidney transplantation		81 (58.3)	44 (61.1)	37 (55.2)	0.73
Combined pancreas-kidney transplantation		2 (3.0)	0	2 (1.4)	0.14
Preemptive kidney transplantation		7 (5.0)	4 (5.6)	3 (4.5)	0.77
**Previous KT - number of participants (%)**		6 (4.3)	3 (4.2)	3 (4.5)	0.93
**RRT before transplantation - number of participants (%)**					
Hemodialysis (HD)		122 (87.8)	61 (84.7)	61 (91.0)	0.26
Peritoneal dialysis (PD)		10 (7.2)	7 (9.7)	3 (4.5)	0.23
**Coexisting diseases - number of participants (%)**					
Diabetes mellitus		109 (78.4)	56 (77.8)	53 (79.1)	0.85
Hypertension		52 (37.4)	23 (31.9)	29 (43.3)	0.17
Hyperlipidemia		117 (84.2)	59 (81.9)	58 (86.6)	0.46
Chronic liver diseases		7 (5.0)	2 (2.8)	5 (7.5)	0.21
Autoimmune diseases		4 (2.9)	1 (1.4)	3 (4.5)	0.28
Malignancy		7 (5.0)	4 (5.6)	3 (4.5)	0.77
**Mean age of the donor (SD) – years**		37.7 (12.8)	37.2 (13)	38.3 (12.5)	0.61
**HLA Mismatch - Median (IQR)**		3 (2–3)	2 (2–3)	3 (2–3)	0.20
**CMV serostatus of donor and recipients - number of participants (%)**					
Donor positive, recipient positive		132 (95.0)	69 (95.8)	63 (94.0)	0.63
Donor negative, recipient positive		4 (2.9)	2 (2.8)	2 (3.0)	0.94
**Induction therapy - number of participants (%)**					
Basiliximab		72 (51.8)	36 (50.0)	36 (53.7)	0.66
Anti-thymocyte globulin		16 (11.5)	8 (11.1)	8 (11.9)	0.88
Rituximab		1 (0.7)	1 (1.4)	0	0.33
**History of graft rejection - number of participants(%)**		28 (20.1)	11 (15.3)	17 (25.4)	0.14
**Laboratory parameters - median (IQR)**					
White blood cell count, 10^3^/mcL		7.63 (6.41–9.43)	7.85 (6.50–9.64)	7.35 (6.41–8.99)	0.68
Absolute lymphocyte count, 10^3^/mcL		2.12 (1.68–2.75)	2.27 (1.82–2.79)	2.02 (1.55–2.47)	0.03
Serum creatinine, mg/dL		1.30 (1.07–1.73)	1.23 (1.06–1.61)	1.38 (1.09–2.04)	0.07
**Mean duration between the second vaccine dose and the anti-spike RBD testing – day**		35 (30–40)	35 (30–39)	35 (30–40)	0.87
**Level of anti-spike RBD IgG 4-week post-second vaccine dose – median (IQR)**					
Anti-spike RBD IgG level, AU/mL^a^		56.6 (4.9–465.8)	449.6 (131.2-1172.1)	4.6 (2–14.8)	**< 0.001**
Anti-spike RBD IgG level, BAU/mL^b^		8.04 (0.7–64.87)	63.84 (18.6–166.4)	0.7 (0.3–2.1)	**< 0.001**

Abbreviations: KTR, kidney transplant recipient; KT, kidney transplantation; RRT, renal replacement therapy; HLA, human lymphocyte antigen; CMV, cytomegalovirus; IQR, interquartile range; SD, standard deviation; AU, arbitrary units; BAU, binding antibody units.

Conversion factor: To convert AU/mL to BAU/mL, multiply by 0.142.

^a,b^ Positive cut-off values for anti-spike RBD IgG: 50 AU/mL^a^ and 7 BAU/mL.^b^.

**Fig. 1:**
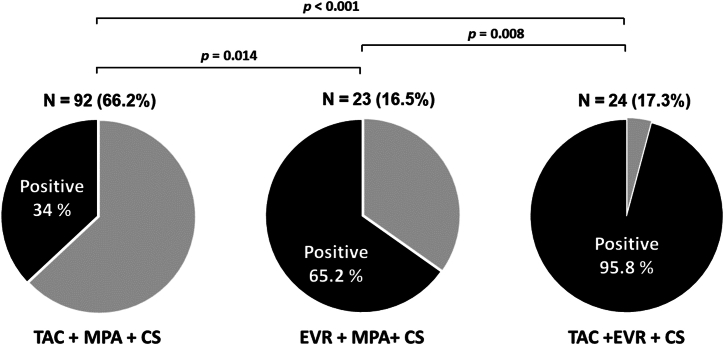
The serological response of anti-spike RBD IgG among the prespecified maintenance immunosuppressant regimen subgroups. Abbreviations: TAC, tacrolimus; EVR, everolimus; MPA, mycophenolic acid.

**Fig. 2:**
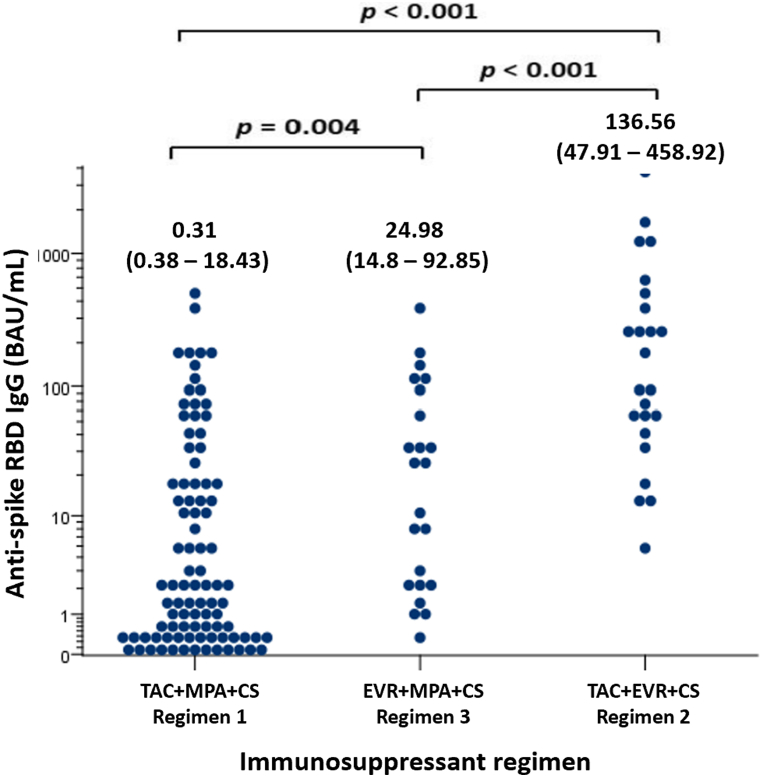
The level of anti-spike RBD IgG among the prespecified maintenance immunosuppressive regimen subgroups (anti-spike RBD IgG, median with IQR). Abbreviations: TAC, tacrolimus; EVR, everolimus; MPA, mycophenolic acid; CS, prednisolone. Positive cut-off values: 50 AU/mL or 7 BAU/mL.

The prevalence of seropositivity assessed by anti-RBD after receiving two doses of vaccine was 51.8 %. The median level of anti-RBD was 8.04 (0.7–64.87) BAU/mL. The prevalence of antibody detection was significantly higher in KTRs who received TAC + EVR + CS (Regimen 2) than EVR + MPA + CS (Regimen 3) and TAC + MPA + CS (Regimen 1), respectively (95 % vs. 65 % vs. 34 %; *p* < 0.001) (Fig. 1). The prescription of the MPA-containing regimen was associated with a suboptimal immune response (OR 0.02, 95%CI 0.01–0.16; *p* < 0.001), while patients who received the EVR regimen had a higher seropositivity rate compared to those on other regimens (OR 12.97, 95%CI 4.69–35.84; *p* < 0.001). The median level of anti-RBD was significantly higher in patients who received TAC + EVR + CS (Regimen 2) than EVR + MPA + CS (Regimen 3) (136.56 (47.91–458.92) vs 24.98 (14.8–92.85) BAU/mL; *p* < 0.001) and TAC + MPA + CS (Regimen 1), respectively (136.56 (47.91–458.92) vs 0.31 (0.38–18.43) BAU/mL; *p* < 0.001). The median antibody level in patients with EVR + MPA + CS (Regimen 3) was significantly higher than that of TAC + MPA + CS (Regimen 1) (*p* = 0.004) (Fig. 2).

The logistic regression analysis revealed the factors associated with seropositive anti-RBD, as shown in Table 2. From the multivariate analysis, patients with an estimated glomerular filtration rate (eGFR) ≥ 60 mL/min/1.73 m^2^ had a significantly higher prevalence of seropositivity after vaccination (OR 2.73, 95%CI 1.09–6.83; *p* = 0.03). Receiving MPA was significantly associated with the absence of an antibody response to vaccination (OR 0.02, 95%CI 0.01–0.16; *p* < 0.001). However, patients who received a daily dose of MPA ≤1000 mg tended to have a better response (*p* < 0.09), although this was not statistically significant.

**Table 2:** Factors associated with the seropositivity of anti-spike RBD IgG after receiving two doses of the ChAdOx1 nCoV-19 vaccine.

Variables	Univariate analysis			Multivariate analysis		
	OR	95 % CI	p-value	OR	95 % CI	p-value
Age	0.99	0.95–1.01	0.23			
Gender (male)	1.19	0.59–2.39	0.62			
Time from transplantation	1.00	1.00–1.01	0.61			
Co-existing diabetes mellitus	0.93	0.41–2.08	0.85			
Co-existing hypertension	0.62	0.31–1.23	0.17			
History of kidney graft rejection	0.53	0.23–1.24	0.14			
White blood cell counts	1.00	1.00–1.00	0.37			
Absolute lymphocyte counts	1.00	1.00–1.01	0.03	1.00	1.00–1.00	0.09
eGFR ≥60 mL/min/1.73 m2	2.22	1.13–4.39	0.02	2.73	1.09–6.83	0.03

Immunosuppressant regimen						
TAC/MPA/CS	Reference			Reference		
EVR/MPA/CS	3.20	1.23–8.33	0.01	4.52	1.43–14.30	0.01
TAC/EVR/CS	39.2	5.0–303.7	< 0.001	104.0	10.7–1013.8	< 0.001

Specific immunosuppressive agents						
MPA-used	0.03	0.01–0.25	0.001	0.02	0.01–0.16	< 0.001
MPA dose ≤1000 mg/day	1.06	0.48–2.35	0.09			
TAC-used	0.52	0.20–1.31	0.16			
TAC level ≤3 ng/mL	6.06	1.27–29.05	0.02	7.64	1.48–39.58	0.02
EVR-used	7.20	3.11–16.70	< 0.001	12.97	4.69–35.84	< 0.001

Abbreviations: OR, odds ratio; CI, confident interval; TAC, tacrolimus; EVR, everolimus; MPA, mycophenolic acid; CS, prednisolone eGFR, estimated glomerular filtration rate.

Immunosuppressive factors associated with seropositivity were the EVR-containing regimen (OR 12.97; 95%CI 4.69–35.84; *p* < 0.001) and a tacrolimus trough level of less than 3 ng/mL (OR 7.64; 95%CI 1.48–39.58; *p* = 0.02).

For the level of anti-RBD after vaccination, we found that age and immunosuppressive regimens were significant factors affecting antibody levels in kidney transplant recipients. Younger age and the use of the TAC/EVR/CS regimen were associated with significantly enhanced immune responses (Supplementary Table 3).

**Fig. 3:**
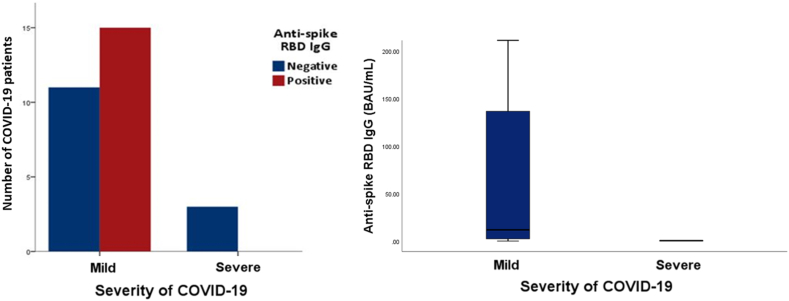
Association between serological response and anti-spike RBD IgG level with the severity of COVID-19 (anti-spike RBD IgG, median with IQR). Positive cut-off values: 50 AU/mL or 7 BAU/mL∗.

During a monitoring period of 11.4 ± 0.8 months for SARS-CoV-2 infection, 29/139 patients (20.9 %) were confirmed to have SARS-CoV-2 infection. Among them, the majority (26/29; 90 %) developed mild or asymptomatic SARS-CoV-2 infection, while only 3/29 patients (10.3 %) experienced severe SARS-CoV-2 infection, all of whom were in the seronegative group and received TAC + MPA + CS. KTRs who experienced severe COVID-19 had significantly lower mean anti-RBD level at 1 month post the second dose of vaccine than those who had a mild infection (p = 0.038), as shown in Fig. 3.

The incidence of overall SARS-CoV-2 infection was lowest in TAC + EVR + CS group (12.5 %), followed by TAC + MPA + CS (21.7 %) and EVR + MPA + CS (30.4 %), with no statistically significant differences. The Kaplan-Meier survival for all types of SAR-CoV-2 infection across immunosuppressive regimens also showed no significant differences in the time to infection (Supplementary Fig. 2).

**Fig. 4:**
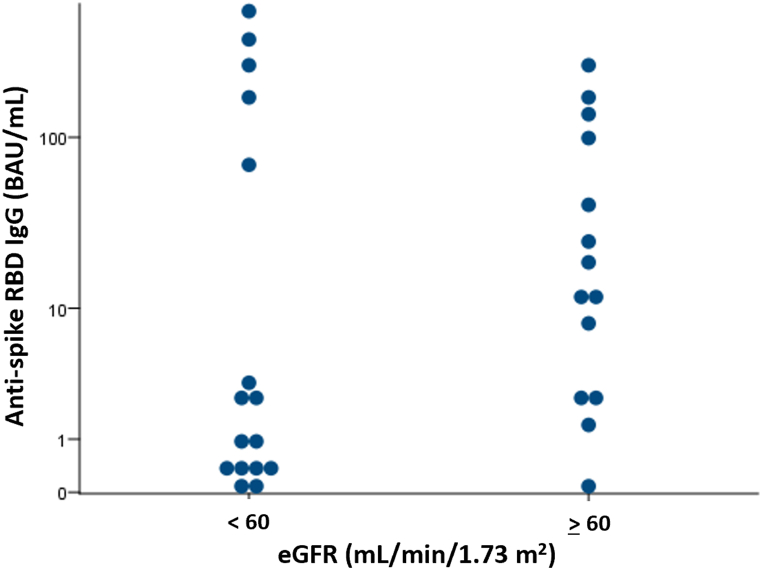
Comparison of anti-RBD levels after receiving a third dose of the mRNA platform vaccine between kidney transplant recipients (KTRs) with eGFR ≥60 and < 60 mL/min/1.73 m^2^ (anti-spike RBD IgG, median with IQR). Abbreviations: eGFR, estimated glomerular filtration rate. Positive cut-off values: 50 AU/mL or 7 BAU/mL∗.

Among the seronegative group, 19 out of 33 (57.6 %) patients tested seropositive four weeks after receiving a third dose of the mRNA platform vaccine (either BNT162b2 from Pfizer BioNTech® or mRNA-1273 from Moderna®). The median antibody level after the third vaccine dose was 36.76 (23.1–111.48) BAU/mL. The median level of anti-RBD was higher in patients with eGFR ≥60 mL/min/1.73 m^2^ than in those with eGFR <60 mL/min/1.73 m^2^ (50.27 (14.06–262.27) vs 4.82 (0.57–25.13) BAU/mL; *p* = 0.02) (Fig. 4).

## 5 Discussion

The suboptimal immune response to vaccination in KTRs who received lifelong immunosuppression has previously been recognized in studies involving various types of vaccines, including those for hepatitis B virus, influenza, herpes zoster, and COVID-19 [4,19,20]. Our prospective cohort study assessed humoral immune response after two doses of the ChAdOx1 nCoV-19 vaccine in Thai KTRs who were naive to vaccination or infection. The seropositive rate for anti-RBD in our KTRs was 51.8 %, which was lower than that reported in studies among healthy populations (100 %) and dialysis patients (94.2 %) [19,21]. However, it is crucial to consider graft function at the time of vaccination. KTRs with better kidney function exhibited a higher serological response compared to those with an eGFR <60 mL/min/1.73 m^2^, which is consistent with previous studies involving inactivated and mRNA vaccines [10].

There is limited evidence regarding the impact of overall immunosuppressive regimens, encompassing not only specific agents but also their combined effects, on the antibody response after vaccination. Our studies revealed that TAC + EVR + CS (Regimen 2) had the best immunologic response in terms of both seropositive rate and antibody level, compared to the other regimens. The seropositivity rate following two doses of ChAdOx1 in the TAC + EVR + CS (Regimen 2) group (95.8 %) is relatively comparable to that observed in the general population who received the same dose and schedule (100 %) [19,22]. In our multivariable analysis, which adjusted for key factors, the TAC + EVR + CS regimen remained superior in achieving higher anti-RBD antibody levels, further supporting its beneficial impact on vaccine response.

The TRANSFORM study demonstrated the benefits of the de novo use of TAC + EVR + CS in reducing the incidence of BK polyoma viral and cytomegaloviral infections after transplantation without increasing early rejection or graft failure rates [23]. The target trough levels of TAC and EVR maintained in our study (Supplement Table 2) were equivalent to those in the TRANSFORM study [23]. A favorable vaccine response is another benefit of the TAC + EVR + CS regimen (Regimen 2). However, due to its relatively weaker immunosuppression, the long-term outcomes, particularly concerning the development of donor-specific HLA antibodies and late antibody-mediated rejection, remain to be determined.

The current standard regimen consisting of TAC + MPA + CS (Regimen 1) provided the lowest humoral response. The use of MPA was strongly associated with low seropositivity following COVID-19 vaccination, consistent with previous study [9]. Similarly, the negative impact of the standard regimen on vaccine response in KTR was also observed in other COVID-19 vaccine platforms, including mRNA (seropositive rate 24–67 %) [9,21,24] and another viral vector vaccine, Ad26.CoV2.S (Johnson & Johnson) (seropositive rate 32 %) [25]. The daily dose of MPA showed an inverse correlation with the vaccine response. KTRs who received daily doses of MPA <1000 mg exhibited a better antibody response, consistent with findings from a previous study [26]. It was found that temporarily suspending MPA during the vaccination period significantly increased the seroconversion rate of anti-RBD compared to those in whom MPA was continued (75 vs 46 %, p < 0.05) [14]. However, some studies demonstrated strategies to stimulate the immune response in seronegative kidney transplant recipients following mRNA vaccination by withdrawing MPA. The results showed no significant increase in seropositivity. Therefore, the impact of MPA withdrawal on enhancing the immune response remains inconclusive [27]. Moreover, the development of donor-specific antibodies and acute cellular rejection in few patients who stopped MPA could discourage the application of this strategy in routine clinical practice [14].

The use of mTORi has been shown to have a positive effect on the antibody response after vaccination. In our study, the substitution of TAC or MPA with EVR had a better response than the regimens without EVR. This finding is consistent with a previous study based on the BNT162b2 vaccine [28]. This may be explained by the ability of mTORi to modulate key immune functions, including T-cell activation, dendritic cell function, and B-cell antibody production, while reducing the suppressive activity of regulatory T cells [29]. Therefore, the effect of mTORi on immune responses after vaccination is attributed to their ability to enhance the activation and function of immune cells, while maintaining a balance that avoids excessive immune suppression. The enhanced response observed with EVR-containing regimens may be due to from EVR alone or the combined effect of lowering TAC, which helps preserve T and B cell function critical for antibody production. Overall, mTORi offer a more favorable immune response compared to other immunosuppressive medications. This findings are consistent with prior study showing that periodic switching from MPA to mTORi for 2 weeks before and after receiving a booster dose of the BNT162b2 vaccine, in addition to two doses of the ChAdOx1 nCoV-19 vaccine, led to significantly higher levels of antibodies against COVID-19 infection, compared to KTRs who remained on their previous regimen [30]. However, the requirement for additional drug level monitoring and the lack of data on long-term immunologic complications might limit the use of this strategy.

Our study highlights the crucial role of vaccination in preventing severe COVID-19 infections among KTRs. None of the KTRs who tested seropositive after vaccination experienced severe COVID-19 infection. However, among the non-responder group, 3 patients (4.1 %) suffered from COVID-19 pneumonia. Given the relatively small sample size, confirming the significance of antibody response may be challenging. Therefore, administering an additional booster dose of the COVID-19 vaccine could be pivotal in stimulating immune response in KTRs. Notably, more than half of the seroconversions occurred following the administration of the third COVID-19 vaccine dose. For a few non-responding KTRs, additional doses beyond the third might be necessary.

The viral vector COVID-19 vaccine serves as an alternative for patients who experienced allergic reactions or complications following the commonly used mRNA vaccine. In our study, KTRs with favorable graft function, who were on long-term TAC + EVR + CS therapy (Regimen 2), exhibited a seropositivity rate comparable to those of the healthy population [19,22]. However, regimens containing MPA demonstrated insufficient antibody response. Monitoring of antibody levels and consideration of additional doses may be necessary for non-responders among KTRs who received MPA-based protocols, while such measures might not be required for those on TAC + EVR + CS regimens. Nonetheless, the selection of maintenance immunosuppression should be carefully weighed in light of both immunologic risk and patient profiles, rather than solely based on antibody response to vaccination.

Our study has some limitations. Firstly, anti-spike RBD IgG might not be the gold standard for assessing the immune response. There is no identified universal level for prevention against infection, as it varies among SARS-CoV-2 variants. The RBD of the Spike protein of SARS-CoV-2 is the specific region where the virus directly interacts with the ACE2 receptor of the host cell. Therefore, the RBD of the SARS-CoV-2 spike protein is the primary target of neutralizing antibodies. A higher level of anti-RBD antibody potentially correlates with the prevention of infections, and the test can be performed more easily and economically than neutralizing antibody assays [16]. Secondly, prior SARS-CoV-2 infection in our study was defined based on self-reported symptomatic infection, without assessing baseline anti-RBD levels or N protein. However, given the extremely low prevalence of SARS-CoV-2 at the time of initiating vaccination in Thailand, with less than 30,000 reported cases, representing only 0.04 % of the entire population. Consequently, the likelihood of acquiring an infection before enrollment was exceedingly low [31]. Thirdly, we did not assess the cellular response following vaccination, nor did we conduct a direct comparison to a healthy control group. Fourthly, we have limited data on additional COVID-19 vaccines administered during the follow-up period, which could interfere with the interpretation of the relationship between the serological response of anti-RBD and the prevalence of COVID-19 infection. The incidence of overall and serious infections was too low to demonstrate significant differences in the protective ability of vaccine across immunosuppressive regimens. Finally, the study did not consider the impact of SARS-CoV-2 strain dynamics during the study period due to a lack of data. However, our study directly compared the antibody response after vaccination among each immunosuppressive regimen, rather than focusing solely on specific agents.

## 6 Conclusion

The anti-RBD response after ChAdOx1 nCoV-19 vaccination was observed in 51.8 % of the KTRs. Patients who received the TAC/EVR/CS regimen exhibited the highest immune response after vaccination, which was relatively comparable to that seen in the general population. The immunosuppressive regimen should be taken into consideration for administering an additional vaccine dose in KTRs.

## CRediT authorship contribution statement

**Nuttasith Larpparisuth:** Writing – review & editing, Writing – original draft, Visualization, Validation, Supervision, Software, Resources, Project administration, Methodology, Investigation, Funding acquisition, Formal analysis, Data curation, Conceptualization. **Kritsada Pongsakornkullachart:** Writing – original draft, Visualization, Validation, Software, Resources, Methodology, Investigation, Formal analysis, Data curation, Conceptualization. **Nartsiri Ratchawang:** Methodology, Investigation, Data curation. **Attapong Vongwiwatana:** Writing – review & editing, Supervision, Project administration, Methodology, Investigation, Formal analysis, Conceptualization. **Peenida Skulratanasak:** Writing – review & editing, Writing – original draft, Visualization, Validation, Supervision, Software, Resources, Project administration, Methodology, Investigation, Funding acquisition, Formal analysis, Data curation, Conceptualization.

## Data availability

The datasets that support the findings of this study are available from the corresponding author upon reasonable request.

## Ethical declarations

This study was approved by the Siriraj Institutional Review Board (COA no. Si 928/2021). All participants provided informed consent to participate in the study. The study was performed in accordance with international guidelines for human research protection.

## Funding

Routine to research unit (R2R) of Siriraj hospital, 10.13039/501100004156Mahidol University (R2R.635/22)

## Declaration of competing interest

The authors declare that they have no known competing financial interests or personal relationships that could have appeared to influence the work reported in this paper.
