# Supplementary material for: Impact of immunosuppressive regimens on antibody response after COVID-19 vaccination among Thai kidney transplant recipients
Source: Heliyon. 2025 Jan 25;11(3):e42291. doi: 10.1016/j.heliyon.2025.e42291 (PMC11808495; doi:10.1016/j.heliyon.2025.e42291)
Supplement: Multimedia component 1 [file mmc1.docx]

**Supplementary Material**

Impact of immunosuppressive regimens on antibody response after COVID-19 vaccination among Thai kidney transplant recipients

**Supplementary Figure 1:** Consort flow diagram of the study

140 eligible KTRs were screened.

1 Excluded: Incomplete antibody testing

139 KTRs were included in the primary analysis.

33 KTRs received 3^rd^ dose of vaccine and 2^nd^ test of anti-spike RBD IgG.
(Non-responder subgroup)

**SupplementaryTable 1:** The dosage and levels of each maintenance immunosuppressants at the time of vaccination, stratified according to anti-spike RBD IgG status after receiving two doses of the ChAdOx1 nCoV-19 vaccine

| **Characteristics** | **Total  (N=139)** | | **Anti-spike RBD IgG** | | | | |
| --- | --- | --- | --- | --- | --- | --- | --- |
|  |  |  | **Seropositive (N=72)** | | **Seronegative (N=67)** | | ***p*-value** |
|  | No. | Values | No. | Values | No. | Values |  |
| **Dosage of immunosuppressants – median (IQR)** | | | | | | | |
| Tacrolimus, mg/day | 116 | 2.00 (1.38-2.75) | 57 | 1.75 (1.00-2.50) | 59 | 2.00 (1.50-3.00) | 0.047 |
| Everolimus, mg/day | 47 | 0.75 (0.50-1.00) | 38 | 0.50 (0.50-0.75) | 9 | 1.00 (0.75-1.00) | 0.022 |
| Mycophenolic acid^a^, mg/day | 115 | 1000 (750-1500) | 49 | 1000 (1000-1250) | 66 | 1000 (750-1500) | 0.66 |
| Prednisolone, mg/day | 139 | 5 (5-5) | 72 | 5 (5-5) | 67 | 5 (5-5) | N/A |
| **Level of immunosuppressants – mean ± SD** | | | | | | | |
| Tacrolimus trough level, ng/mL | 116 | 5.46 ± 1.82 | 57 | 4.99 ± 1.80 | 59 | 5.92 ± 1.74 | 0.005 |
| Everolimus trough level, ng/mL | 47 | 5.32 ± 1.38 | 38 | 5.08 ± 1.37 | 9 | 6.33 ± 0.95 | 0.01 |

Abbreviations: IQR, interquartile range; SD, standard deviation; N/A, not applicable

^a^Mycophenolic acid 500 mg is equivalent to mycophenolate sodium 360 mg

**Supplementary Table 2:** The maintenance immunosuppressants in the prespecified immunosuppressive regimens subgroup

| **Immunosuppressants** | **Total  (N=139)** | | **Anti-spike RBD IgG** | | | | |
| --- | --- | --- | --- | --- | --- | --- | --- |
|  |  |  | **Seropositive (N=72)** | | **Seronegative (N=67)** | | ***p*-value** |
|  | No. | Values | No. | Values | No. | Values |  |
| **TAC/MPA/CS regimen** | | | | | | | |
| TAC, C_0_ level - mean ± SD, ng/mL | 92 | 6.02 ± 1.56 | 34 | 6.16 ±1.20 | 58 | 5.94 ± 1.75 | 0.56 |
| Dosage of MPA - median (IQR), mg/day | 92 | 1000 (1000-1500) | 34 | 1000 (1000-1500) | 58 | 1000 (750-1500) | 0.97 |
| **EVR/MPA/CS regimen** | | | | | | | |
| EVR, C_0_ level - mean ± SD, ng/mL | 23 | 6.27 ± 1.03 | 15 | 6.17 ± 1.09 | 8 | 6.44 ±0.94 | 0.50 |
| Dosage of MPA - median (IQR), mg/day | 23 | 1000 (750-1250) | 15 | 1000 (750-1250) | 8 | 1000 (875-1000) | 0.89 |
| **TAC/EVR/CS regimen** | | | | | | | |
| TAC, C_0_ level - mean ± SD, ng/mL | 24 | 3.31 ± 0.93 | 23 | 3.26 ± 0.91 | 1 | 4.60 | 0.17 |
| EVR, C_0_ level - mean ± SD, ng/mL | 24 | 4.41 ± 1.02 | 23 | 4.37 ± 1.02 | 1 | 5.39 | 0.17 |

Abbreviations: TAC, tacrolimus; EVR, everolimus; MPA, mycophenolic acid; CS, prednisolone, C_0_ level, trough level; IQR, interquartile range; SD, standard deviation

^a^Mycophenolic acid 500 mg is equivalent to mycophenolate sodium 360 mg

**Supplement Table 3**: Factors associated with the level of anti-spike RBD IgG (BAU/mL) after receiving two doses of the ChAdOx1 nCoV-19 vaccine, which was analyzed by a multivariate linear regression analysis.

| **Variables** | **Unstandardized Coefficients (B)** | **Standardized Beta (𝛽)** | **P-value** | **95%Confidence Interval for B** | |
| --- | --- | --- | --- | --- | --- |
|  |  |  |  | **Lower bound** | **Upper bound** |
| Constant | 314.4 |  | .11 | -69.76 | 698.55 |
| **Age (years)**  Gender  female 0, male 1  Time from transplantation (months)  Diabetes mellitus  Hypertension  eGFR (ml/min/1.73m^2^) | -12.15  -17.40  -.91  89.69  83.2  .55 | -.34  -.02  -.12  .09  .10  .03 | **<0.001**  .79  .11  .28  .25  .70 | -18.56  -146.62  -2.045  -72.78  -58.79  -2.23 | -5.75  111.82  .22  252.17  225.2  3.34 |
| **Immunosuppressive regimens**  TAC/MPA/CS (1)  EVR/MPA/CS (2)  TAC/EVR/CS (3) | 224.22 | .42 | **<0.001** | 141.00 | 307.44 |

Abbreviations: TAC, tacrolimus; EVR, everolimus; MPA, mycophenolic acid; CS, prednisolone; eGFR, estimated glomerular filtration rate

**Supplement Figure 2**: Kaplan-Meier survival plot illustrating the overall SARS-CoV-2 infection-free survival among kidney transplant recipients after vaccination, stratified by the immunosuppressive regimens.

**
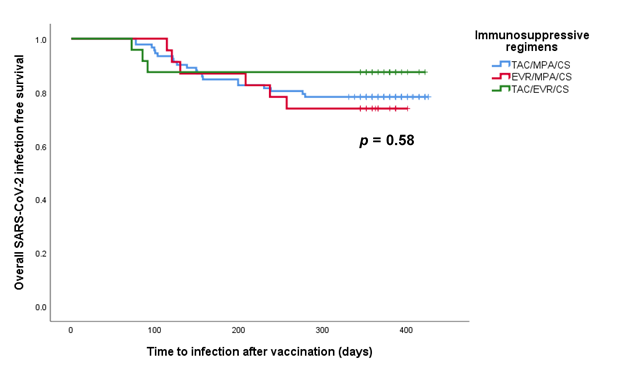
**

Abbreviations: TAC, tacrolimus; EVR, everolimus; MPA, mycophenolic acid; CS, prednisolone
